# The Role of CD40 in Allergic Rhinitis and Airway Remodelling

**DOI:** 10.1155/2021/6694109

**Published:** 2021-04-23

**Authors:** Ke-Jia Cheng, Min-Li Zhou, Yong-Cai Liu, Chen Wang, Ying-Ying Xu

**Affiliations:** Department of Otolaryngology, The First Affiliated Hospital, School of Medicine, Zhejiang University, Hangzhou, China

## Abstract

**Background:**

Allergic rhinitis (AR) affects millions of people and is lack of effective treatment. CD40 is an important costimulatory molecule in immunity. However, few studies have focused on the role of CD40 in AR.

**Methods:**

In this study, we built mouse model of chronic AR. The mice were divided into the AR, control, intravenous CD40 siRNA, and nasal CD40 siRNA groups (*n* = 6 each). We detected OVA-sIgE, IL-4, IL-5, IL-13, IL-10, IFN-*γ*, and TGF-*β* levels in serum and supernatant by ELISA, CD40^+^ splenic DCs, and Foxp3^+^ Tregs by flow cytometry and CD40 mRNA by RT^2^-PCR. We also used PAS and MT stains to assess tissue remodelling.

**Results:**

(1) The OVA-sIgE, IL-4, IL-5, and IL-13 levels in the serum or supernatant of nasal septal membrane of AR mice were significantly higher than control. After treated with CD40 siRNA, those indicators were significantly decreased. The IFN-*γ*, IL-10, and TGF-*β* levels in AR mice were significantly lower than that in control and were increased by administration of CD40 siRNA. (2) AR mice had significantly fewer Foxp3^+^ Tregs in the spleen than control mice. After treated with CD40 siRNA, AR mice had significantly more Foxp3^+^ Tregs. (3) AR mice exhibited a significantly higher CD40 mRNA levels than control. Administration of CD40 siRNA significantly reduced the CD40 mRNA level. (4) The AR mice showed significantly greater collagen deposition than the control in MT staining. Applications of CD40 siRNA significantly reduced the collagen deposition in AR mice.

**Conclusion:**

CD40 siRNA therapy shows promise for chronic AR as it significantly attenuated allergic symptoms and Th2-related inflammation and upregulated Foxp3^+^ Tregs. CD40 plays a role in tissue remodelling in AR, which can be inhibited by CD40 siRNA application.

## 1. Introduction

Allergic rhinitis (AR) affects millions of people and is induced by immunoglobulin E (IgE). AR is a common disorder caused by a type 2 immune response to antigen as well as the recruitment and activation of mast cells, eosinophils, and basophils [1, 2]. Type 2 inflammation promotes the activity of the cytokines IL-4, IL-5, and IL-13 [3]. IL-4 causes isotype class switching in B cells to produce IgE; IL-5 promotes eosinophil activation and recruitment; IL-13 triggers mucus hypersecretion and airway hypersensitivity [4, 5]. AR can be divided into intermittent and persistent subtypes and is characterised by nasal congestion, rhinorrhoea, sneezing, and itching [6]. There is no effective treatment for AR, which tends to recur and impair the quality of life of affected patients.

Airway remodelling is defined as any change in the composition, distribution, thickness, mass, volume, and number of structural components in the airway wall of patients compared with healthy individuals [7]. It usually manifests as goblet cell hyperplasia, basal membrane thickening, subepithelial fibrosis, hyperplasia and hypertrophy of smooth-muscle cells, and angiogenesis [8]. Airway remodelling is common in lower airway diseases, particularly asthma [9]. Asthma is difficult to treat, due in part to airway remodelling. Because of the difference in smooth muscle and blood sinus between the lower and upper airways, whether remodelling involves in AR is still controversial. Some researches have implied the existence of remodelling in AR [10, 11]. Hence, we speculated that refractory AR might be linked to remodelling of the nasal mucosa.

CD40 is a type I membrane protein and a member of the tumour necrosis factor receptor (TNFR) superfamily [12]. It is expressed on B cells, dendritic cells (DCs), macrophages, monocytes, epithelial cells, endothelial cells, mesenchymal cells, and platelets. The CD40 pathway is an important costimulatory pathway in immunity. The interaction of CD40 and its ligand is required for effective adaptive and innate immune responses as it induces several immunostimulatory events, including the licensing of antigen-presenting cells (APCs), T cell activation, and B cell class switching [13]. CD40 has been linked to numerous diseases, including metabolic, neoplastic, autoimmune, cardiovascular, bowel, and allergic conditions. Moreover, CD40 is implicated in lower airway diseases, especially in allergy. However, few studies have focused on the role of CD40 in AR.

In the present study, we investigated the feasibility of CD40 small interfering RNA (siRNA) as a means of controlling AR and related airway remodelling *in vivo*. We found that nasal application of CD40 siRNA is effective against chronic AR and airway remodelling.

## 2. Materials and Methods

### 2.1. Ethics Statement

All mice were managed in accordance with protocols approved by the Research Ethics Committee of the First Affiliated Hospital, College of Medicine, Zhejiang University (Zhejiang, China).

### 2.2. Mouse Model of Chronic AR

Six-week-old adult BALB/c mice weighing 18–20 g were purchased from Shanghai Animal Laboratories (Shanghai, China). The mice were divided into the AR, control, intravenous CD40 siRNA, and nasal CD40 siRNA groups (*n* = 6 each). AR mice were injected intraperitoneally with 10 *μ*g ovalbumin (OVA) and 4 mg Al(OH_3_) on days 0 and 14 and challenged intranasally on days 29 to 35 with OVA (600 *μ*g); thereafter, the mice were challenged intranasally twice weekly for 8 weeks. Subsequently, sneezing and nasal rubbing movements were evaluated for 30 min. Control mice underwent intranasal application of phosphate-buffered saline (PBS) only. The mice were sacrificed 24 h after the last challenge by intraperitoneal injection of excess pentobarbital, and the serum and spleen were collected for analysis. One side of the nasal septal membrane was collected for reverse transcription and real-time polymerase chain reaction (RT^2^-PCR), and the other side was collected for histological assessment.

### 2.3. CD40 siRNA and Gene Silencing

**Figure 1:**
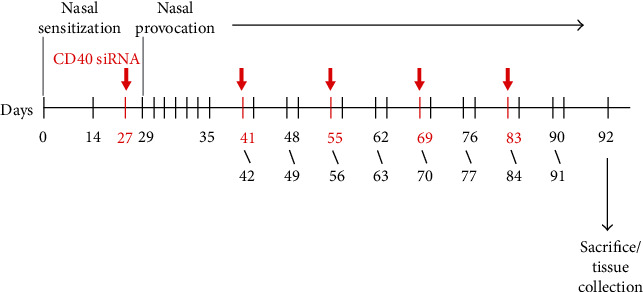
Mouse model of chronic AR and CD40 siRNA application.

The siRNA for silencing of murine CD40 (SI02739016 [FlexiTube siRNA]) was purchased from Qiagen (Qiagen Inc., Germantown, USA). Mice in the intravenous CD40 siRNA group were injected intravenously with 50 *μ*g of CD40 siRNA on days 27, 41, 55, 69, and 83. Mice in the nasal CD40 siRNA group received 60 *μ*g CD40 siRNA intranasally on days 27, 41, 55, 69, and 83. Mice in the treatment and control groups received 60 *μ*g PBS intranasally at the same time points (Figure 1).

### 2.4. Measurement of OVA-sIgE and Inflammatory Cytokine Levels in Serum and Supernatant

Samples were allowed to clot in a serum separator tube for 30 min, centrifuged for 15 min at approximately 1,000 rpm, and stored at −20°C. The nasal septal membrane was collected immediately after euthanasia. The specimens were thawed in saline (0.1 g in 1 mL) and processed in a tissue homogeniser. Next, the suspensions were centrifuged for 5 min at 3,000 rpm. The supernatants were collected and stored at −20°C prior to analysis.

The levels of OVA-sIgE (Bio-Swamp, Wuhan, China), IL-5 (eBioscience, California, USA), and IFN-*γ* (eBioscience, California, USA) in the serum and supernatant were measured by enzyme-linked immunosorbent assay (ELISA) according to the manufacturer's instructions. IL-4 (eBioscience, California, USA), IL-13 (eBioscience, California, USA), IL-10 (eBioscience, California, USA), and TGF-*β* (R&D Systems, Minnesota, USA) levels were also detected by ELISA. In brief, the samples (40 *μ*L) were added to 96-well plates, followed by the appropriate antibody (10 *μ*L). The plates were then closed and incubated for 30 min at 37°C. Wash buffer was added to each well, the plates were allowed to stand for 30 s, and the wash buffer was gently tapped out; this procedure was performed a total of five times. Next, 50 *μ*L of horseradish peroxidase- (HRP-) conjugate reagent were added to each well, except the blank well, and the plate was incubated and washed as before. Chromogen solution A and chromogen solution B (50 *μ*L each) were added to each well, and the plates were gently mixed and incubated for 15 min at 37°C. Next, 50 *μ*L of stop solution were added to each well, and the optical density at 450 nm was read within 15 min. A standard curve was generated to enable quantification.

### 2.5. Preparation of Spleen Mononuclear Cell Suspensions

The minced spleen tissues of BALB/c mice were mixed with RMPI-1640 medium and centrifuged for 10 min at 3,000 rpm. The intermediate white film was next centrifuged in RMPI-1640 medium for 10 min at 3,000 rpm. Finally, red cells were removed using ACK lysis buffer, and the resulting spleen mononuclear cell (sMNC) suspensions were centrifuged in RMPI-1640 medium for 10 min at 3,000 rpm.

### 2.6. Flow Cytometry Analysis of CD40^+^ Splenic DCs and Forkhead Box P3 (Foxp3)^+^ Tregs

sMNC suspensions were diluted with PBS to 10^6^cells/mL. sMNCs (100 *μ*L) were incubated with 5 *μ*L of anti-CD4 and -CD25 mAbs (eBioscience) at 4°C in the dark for 60 min. Then, we fixed and broken membrane. Next, sMNCs were added to 5 *μ*L of anti-Foxp3 mAb (eBioscience). Flow cytometric analysis of Foxp3^+^ Tregs was performed using a FACScan (Becton Dickinson, New Jersey, USA). We used 3 *μ*L of anti-CD40 and 1 *μ*L of anti-CD11c mAb (eBioscience) to analyse CD40^+^ DCs.

### 2.7. RT^2^-PCR for CD40

Total RNA was isolated from nasal septal membranes using TRIzol reagent (Invitrogen, California, USA) according to the manufacturer's protocol. To generate first-strand cDNA, samples were treated with DNase I to remove DNA and subjected to reverse transcription in reactions containing RNA-Primer Mix (5 *μ*L), 25 mM dNTPs (1 *μ*L), 25 U/*μ*L RNase inhibitor (1 *μ*L), 200 U/*μ*L M-MLV reverse transcriptase (1 *μ*L), oligo (1 *μ*L), and DNase-free water (4 *μ*L).

The CD40 primers were sense 5′-CCCTGCGATGGTGTCTTTGC-3′ and antisense 5′-TGGCTTGTCAGTCGGCTTCC-3′. The primers for GAPDH (control) were sense 5′-ATCACTGCCACCCAGAAG-3′ and antisense 5′-TCCACGACGGACACATTG-3′. Real-time PCR was performed at 95°C for 10 min, 95°C for 15 s, 60°C for 45 s (40 cycles), 95°C for 15 s, 60°C for 1 min, 95°C for 15 s, and 60°C for 15 s.

### 2.8. Assessment of Tissue Remodelling

Nasal septal membranes were inflated with 4% paraformaldehyde at 25 cm H_2_O pressure. Paraffin-embedded sections (4 *μ*m) were stained with periodic acid–Schiff (PAS) to identify goblet cell hyperplasia, which was quantified in three selected areas at a 200-fold magnification. The number of goblet cells in the epithelium was separately assessed by two researchers, and the average values were calculated.

The sections were stained with Masson's trichrome (MT) to identify collagen deposition. As previous article described, the area of collagen deposition (blue staining) beneath the basement membrane in three regions was evaluated at a 200-fold magnification. Collagen deposition was evaluated as the percentage blue-stained area in the nasal mucosa using the Image J software [5].

### 2.9. Statistical Analysis

Values are expressed as median (25–75%) using the SPSS version 20.0 software. Differences in symptoms were analysed by Fisher's exact test. Differences between groups were evaluated with the Mann–Whitney *U*-test. A value of *P* < 0.05 was considered indicative of statistical significance.

## 3. Results

### 3.1. CD40 siRNA Treatment Decreases Symptoms in AR Mice

After the last nasal challenge, AR, but not control, mice showed sneezing and nasal rubbing. There was significantly difference between AR and control group. One of the mice administered CD40 siRNA intravenously and nasally showed sneezing and nasal rubbing. There was significantly difference between CD40 siRNA and AR group.

### 3.2. CD40 siRNA Treatment Decreases the OVA-sIgE Level, Inhibits the Th2 Immune Response, and Promotes the Th1 Immune Response

**Figure 2:**
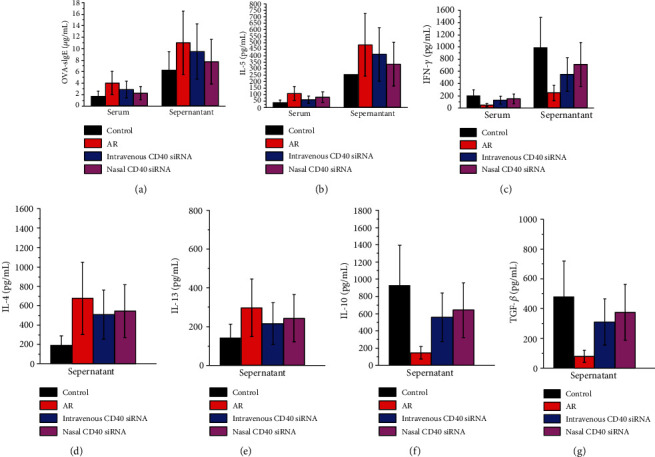
(a) Serum and supernatant levels of OVA-sIgE were measured by ELISA. AR mice produced significantly more OVA-sIgE than control. OVA-sIgE level in mice treated with CD40 siRNA was lower than AR mice. Mice treated nasally with CD40 siRNA produced significantly less OVA-sIgE than mice given intravenously CD40 siRNA. (b) Serum and supernatant levels of IL-5 were measured by ELISA. AR mice produced significantly more IL-5 than control. IL-5 level in mice treated with CD40 siRNA was lower than AR mice. (c) Serum and supernatant levels of IFN-*γ* were measured by ELISA. AR mice produced significantly less IFN-*γ* than control. IFN-*γ* level in mice treated with CD40 siRNA was higher than AR mice. Mice treated nasally with CD40 siRNA produced significantly more IFN-*γ* than mice given intravenously CD40 siRNA. (d) Supernatant levels of IL-4 were measured by ELISA. AR mice produced significantly more IL-4 than control. IL-4 level in mice treated with CD40 siRNA was lower than AR mice. (e) Supernatant levels of IL-13 were measured by ELISA. AR mice produced significantly more IL-13 than control. IL-13 level in mice treated with CD40 siRNA was lower than AR mice. (f) Supernatant levels of IL-10 were measured by ELISA. AR mice produced significantly less IL-10 than control. IL-10 level in mice treated with CD40 siRNA was higher than AR mice. (g) Supernatant levels of TGF-*β* were measured by ELISA. AR mice produced significantly less TGF-*β* than control. TGF-*β* level in mice treated with CD40 siRNA was higher than AR mice. *P* values <0.05 were considered as significance.

Serum and supernatant levels of OVA-sIgE, Th2 cytokine IL-5, and Th1 cytokine IFN-*γ* of AR mice were measured by ELISA. The OVA-sIgE and IL-5 levels in the serum and supernatant of AR mice were significantly higher than those in control mice. The OVA-sIgE and IL-5 levels in mice treated with CD40 siRNA were lower than those in AR mice. Mice treated nasally with CD40 siRNA produced significantly less OVA-sIgE than mice intravenously administered CD40 siRNA (Figures 2(a) and 2(b)). The IFN-*γ* level in the serum and supernatant of AR mice was significantly lower than that in control mice and was increased by administration of CD40 siRNA. The IFN-*γ* level in mice treated nasally with CD40 siRNA was significantly higher than that in mice treated intravenously (Figure 2(c)). Supernatant levels of IL-4, IL-13, IL-10, and TGF-*β* were also measured by ELISA. The IL-4 and IL-13 levels in the supernatant of AR mice were significantly increased compared to those in control. After treated with CD40 siRNA, the IL-4 and IL-13 levels in the supernatant were significantly reduced (Figures 2(d) and 2(e)). However, no difference of the IL-4 and IL-13 levels in the supernatant was observed between nasal and intravenous siRNA groups. In addition, the IL-10 and TGF-*β* levels in the supernatant of AR mice were significantly decreased as compared with those in control. After treated with CD40 siRNA, the IL-10 and TGF-*β* levels in the supernatant were significantly upregulated (Figures 2(f) and 2(g)). Similarly, no difference of the IL-10 and TGF-*β* levels in the supernatant was observed between nasal and intravenous siRNA groups. These results suggest that CD40 siRNA inhibits the allergic and Th2 responses and promotes the Th1 response and immune tolerance. CD40 siRNA applied by nasal pathway may more powerful than intravenous pathway.

### 3.3. CD40 siRNA Treatment Inhibits CD40^+^ DCs and Increases the Foxp3^+^ Treg Population in the Spleen

DCs are important APCs that express CD40 [14]. To assess the efficacy of gene silencing by CD40 siRNA *in vivo*, we investigated CD40 expression on DCs by flow cytometry (Table 1). The number of DCs in AR mice was higher than that in control mice but did not differ between AR and siRNA mice. This suggests that DCs play a role in the pathogenesis of AR, and that this role is independent of that played by CD40.

**Figure 3:**
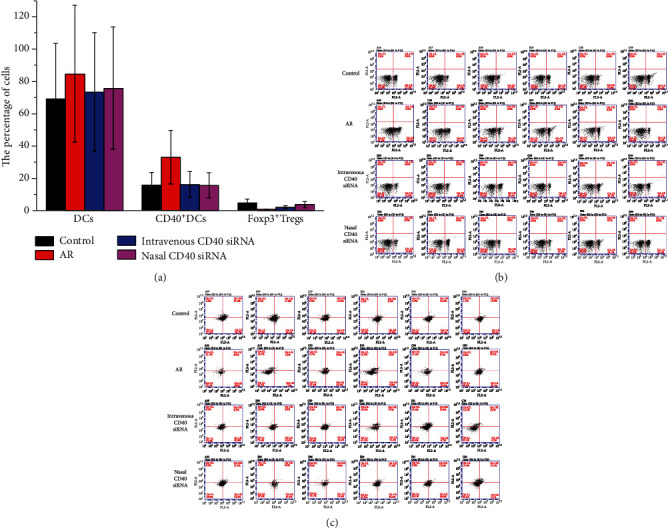
(a) CD40^+^ DCs and Foxp3^+^ Tregs of the spleen in control, AR, intravenous CD40 siRNA, and nasal CD40 siRNA groups were detected by flow cytometry. CD40 expression on DCs in AR mice was higher than control group. There was no difference between AR and siRNA groups. CD40 siRNA treatment resulted in inhibition of CD40 expression on DCs. There was no significant difference between intravenous and nasal siRNA group. AR mice shown significantly less Foxp3^+^ Tregs, compared with control mice. Mice nasally treated with CD40 siRNA had significantly more Foxp3^+^ Tregs, compared with AR mice. There was no significant difference between intravenous CD40 siRNA and AR group. *P* values <0.05 were considered as significance. (b) CD40^+^ DCs of the spleen in control, AR, intravenous CD40 siRNA, and nasal CD40 siRNA groups were detected by flow cytometry. (c) Foxp3^+^ Tregs of the spleen in control, AR, intravenous CD40 siRNA, and nasal CD40 siRNA groups were detected by flow cytometry.

CD40 siRNA treatment suppressed CD40 expression by DCs. There was no significant difference in CD40 expression on DCs between mice treated with the CD40 siRNA intravenously and nasally (Figures 3(a) and 3(b)) Therefore, the CD40 siRNA knocked down CD40 expression by DCs *in vivo*.

Tregs contribute to active immune suppression [15]. We examined the effect of CD40 siRNA on Treg generation *in vivo* by analysing CD4^+^ CD25^+^ Foxp3^+^ cells in sMNCs by flow cytometry (Table 1). AR mice had significantly fewer Foxp3^+^ Tregs than control mice. Mice treated with CD40 siRNA nasally had significantly more Foxp3^+^ Tregs than AR mice. There was no significant difference in the number of Foxp3^+^ Tregs between mice treated intravenously with CD40 siRNA and AR mice (Figures 3(a) and 3(c)). These findings implicate downregulation of Foxp3^+^ Tregs in the pathogenesis of AR and suggest that nasal administration of CD40 siRNA increases the splenic Foxp3^+^ Treg population.

### 3.4. CD40 siRNA Treatment Inhibits CD40 Expression in Nasal Tissues

**Figure 4:**
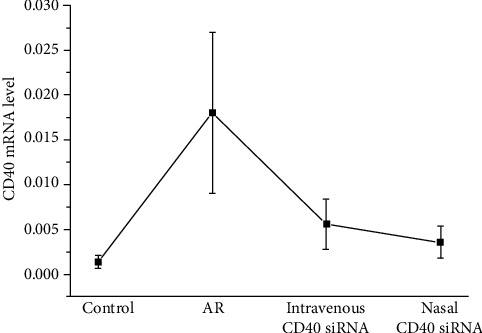
The mRNA level of CD40 in nasal tissues was detected by RT^2^-PCR. AR mice expressed significantly higher CD40 mRNA compared with control group. CD40 mRNA was significantly reduced in the mice treated with CD40 siRNA, both by intravenous and nasal methods. The mice treated with nasal CD40 siRNA expressed significantly lower CD40 mRNA than mice that received intravenous CD40 siRNA. *P* values <0.05 were considered as significance.

The effect of CD40 siRNA on the CD40 mRNA level was evaluated by RT^2^-PCR. AR mice exhibited a significantly higher CD40 mRNA levels than control mice. Intravenous or nasal administration of CD40 siRNA significantly reduced the CD40 mRNA level. The CD40 mRNA level of mice treated with CD40 siRNA nasally was significantly lower than that of mice who received the CD40 siRNA intravenously (Figure 4). This suggests that CD40 plays a role in the pathogenesis of AR, and that CD40 siRNA suppressed CD40 expression *in vivo*.

### 3.5. PAS and MT Staining

**Figure 5:**
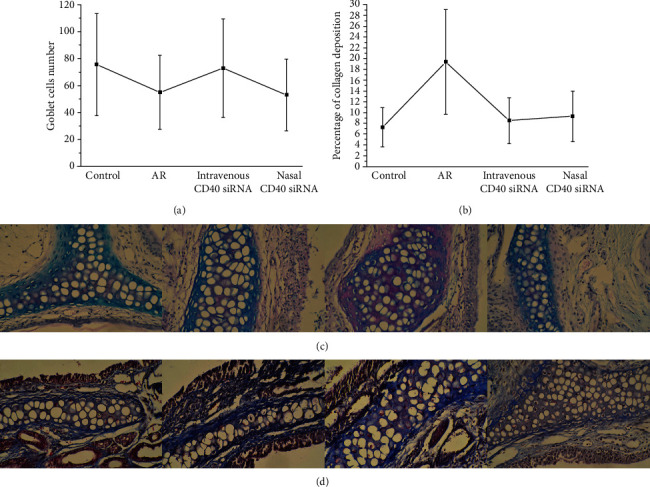
(a) Goblet cell numbers of control, AR, intravenous CD40 siRNA, and nasal CD40 siRNA groups were detected by PAS staining. There was no significant difference in goblet cell numbers between AR, CD40 siRNA treatment, and control groups. All *P* values >0.05. (b) Collagen deposition of control, AR, intravenous CD40 siRNA, and nasal CD40 siRNA groups was detected by MT staining. In this study, AR mice expressed significantly more collagen deposition, compared to control group. Mice treated nasally or intravenously with CD40 siRNA expressed significantly less collagen deposition, compared with AR mice. *P* values <0.05 were considered as significance. (c) Goblet cell numbers of control, AR, intravenous CD40 siRNA, and nasal CD40 siRNA groups were detected by PAS staining. (d) Collagen deposition of control, AR, intravenous CD40 siRNA, and nasal CD40 siRNA groups was detected by MT staining.

Tissue remodelling primarily involves goblet cell hyperplasia and collagen deposition, which are common in lower-airway inflammation, such as asthma [16]. However, whether tissue remodelling occurs in AR is controversial. We enumerated goblet cells by PAS staining and explored collagen deposition by MT staining (Table 2). There was no significant difference in the number of goblet cells among the AR, CD40 siRNA-treated, and control mice (Figures 5(a) and 5(c)). The AR mice showed significantly greater collagen deposition than the control mice. Also, mice treated nasally or intravenously with the CD40 siRNA showed significantly less collagen deposition than did the AR mice (Figures 5(b) and 5(d)). These results suggest that tissue remodelling in AR manifests mainly as collagen deposition that it is related to the CD40 pathway, and that it can be inhibited by CD40 siRNA treatment.

## 4. Discussion

AR, which is Th2-mediated, is an eosinophilic allergic inflammation of the nasal membranes. AR patients have elevated IgE levels, eosinophil infiltration, and type 2 cytokine production [17]. However, the pathogenesis of AR is unclear. Until now, AR mice model usually use an acute model. In the present study, we developed a mouse model of chronic AR in an attempt to more accurately reflect naturally occurring AR. In this study, we found that the levels of OVA-sIgE, IL-4, IL-5, and IL-13 in the serum or supernatant were higher in AR mice than in control mice. Meanwhile, the serum and supernatant IFN-*γ* levels were lower in AR mice. These findings suggest that AR is mainly mediated by Th2 inflammation, which is in accordance with a previous report [18].

The CD40 pathway is an important costimulatory pathway in immunity. The CD40–CD40L interaction leads to several immunostimulatory events, including maturation of APCs, T cell activation, and B cell class switching [12]. Dysfunction of the CD40 pathway is implicated in many diseases, such as neoplastic, allergic, autoimmune, cardiovascular, and bowel disorders. CD40 also plays an important role in allergic inflammation of the lower airway [19–22]. CD40 signalling is important for OVA-induced eosinophilia and the production of IL-4, IL-5, and IL-13 in a mouse model of allergic asthma [19]. CD40 improves the sensitivity of related cells and promotes the production of proinflammatory cytokines after postexercise bronchoconstriction in patients with allergic asthma [20]. Moreover, CD40 induces the expression of numerous signalling molecules associated with airway inflammation and remodelling in allergic asthma [21]. CD40 gene polymorphisms may also influence IgE production in patients with asthma by modulating CD40 expression on B cells [22].

However, there are few publications to show the role of CD40 in AR. Production of specific IgE, IL-4, and IL-5 was reduced in CD40^−/−^ and *Schistosoma mansoni* egg antigen-induced AR mouse models [23]. IgE secretion by B cells was inhibited by promoting the combination of CD40L and IL-4 in patients with AR [24]. Recently, siRNA is used in studies of the role of CD40 in allergic inflammation. siRNA-mediated RNA interference suppresses gene expression *in vitro* and *in vivo* [25] and is effective and long-lasting [26]. Systemic administration of CD40 siRNA reportedly inhibits allergic responses and symptoms *in vitro* and *in vivo* [27, 28]. CD40-silenced DCs have been shown to suppress OVA-induced allergy [29]. Local delivery of siRNA enables use of a lower dose and has a lower incidence of side effects. Therefore, we assessed intravenous and nasal applications of a CD40 siRNA. In this study, intravenous and nasal applications of CD40 siRNA significantly reduced the CD40 mRNA levels in nasal tissues, suggesting silencing of the CD40 gene. Also, the CD40 level differed between AR and control mice, and CD40 siRNA treatment reduced sneezing and nasal rubbing movements by the mice. In addition, CD40 siRNA treatment decreased the OVA-sIgE, IL-4, IL-5, and IL-13 levels and increased the IFN-*γ* level. These findings suggest that CD40 plays an important role in the pathogenesis of AR, and that CD40 siRNA treatment restores the Th1/Th2 balance and inhibits allergic inflammation in AR.

DCs are present in the skin and mucosae [30], and mice have several subgroups of DCs [31]. In this study, we used CD11c^+^ as the screening criterion of DCs by flow cytometry. DCs recognise and capture invading foreign antigens and pathogens and regulate the immune response by interacting with T cells. However, the role of DCs in AR is controversial. DCs may activate effector T cells to promote immune responses or induce Tregs to promote immune tolerance. It has been reported that the number of DCs in the nasal mucosa of patients with AR significantly increases after allergen challenge [32]. In contrast, one study showed that DC-induced generation of IL-10-producing Tregs *in vivo* is dependent on ICOSL/ICOS and results in immune tolerance [33]. Here, we showed that the percentage of DCs in the spleens of AR mice was higher than that in those of control mice, suggesting that DCs promote inflammation in AR. Also, CD40 siRNA treatment significantly reduced CD40 expression on DCs, confirming knockdown of CD40 expression.

Downstream factors in the CD40 pathway are crucial for the maturation of, and cytokine secretion by, DCs. Blockade of CD40-receptor-associated factor-6 impairs DC maturation [34]. Meanwhile, the CD40–CD40L signalling axis induces migration of DCs and reduces the number of Tregs [35]. However, in this study, inhibition of CD40 had no effect on the number of DCs; thus, further research on the relationship between CD40 and DCs is needed.

Treg cells are a CD4^+^ T cell subset that promotes self-immune tolerance and maintenance of immune balance [36]. There are at least two subtypes of Tregs—natural and inducible Tregs [37]. The Foxp3 gene is a critical regulator in the development and function of Tregs. Downregulation of Foxp3 may play an important role in the onset of AR [38]. In this study, the AR mice generated significantly fewer Foxp3+ Tregs, IL-10, and TGF-*β* levels than the control mice, which is in agreement with a previous report [39]. Indeed, CD40 siRNA has been shown to increase Treg numbers in allergic mice [27]. In this study, mice treated nasally, but not those treated intravenously, with CD40 siRNA had significantly more Foxp3^+^ Tregs than AR mice. Meanwhile, after treated with CD40 siRNA, the supernatant IL-10 and TGF-*β* levels were significantly upregulated in AR mice. These results suggest that inhibition of CD40 reduces allergic responses, in part, by increasing the number of Tregs. We postulated that nasal application of CD40 siRNA was more powerful than intravenous.

Tissue remodelling is a common feature of allergic inflammation in the lower airway and can lead to irreversible airflow obstruction [40]. Rhinovirus infection can promote airway remodelling to trigger asthma [41]. However, whether tissue remodelling of the nasal cavity occurs in AR is uncertain. Nasal remodelling, including goblet cell hyperplasia and collagen deposition, has been reported to occur in AR mice [10, 11], whereas other studies have suggested that animal models of AR involve microvascular remodelling of the nasal mucosa [42] and lung remodelling [43]. Additionally, remodelling of the nasal mucosa is reportedly more evident in AR patients with irreversible nasal obstruction [44]. In contrast, Eifan et al. found no histologic features of upper-airway structural remodelling in patients with severe persistent AR [45]. In this study, the goblet cell count was similar in the AR and control mice, but the level of collagen deposition was higher in the AR mice. Thus, it is tempting to speculate that tissue remodelling in AR manifests mainly as collagen deposition. Further studies should focus on the roles of tissue remodelling-related cytokines in chronic AR. CD40 siRNA treatment significantly reduced collagen deposition in AR mice, suggesting that tissue remodelling in AR might be related to the CD40 pathway.

## 5. Conclusions

CD40 siRNA therapy shows promise for chronic AR as it significantly attenuated allergic symptoms and Th2-related inflammation and upregulated Foxp3^+^ Tregs and immune tolerance, which is in agreement with prior research [27, 28]. In this study, we make a new discovery that CD40 siRNA applied by nasal pathway can take roles in antiallergy in chronic AR process. We report here an important role for CD40 in tissue remodelling in AR, which can be inhibited by CD40 siRNA application.

## Figures and Tables

**Table 1:** The percentage of DCs, CD40^+^ DCs, and Tregs in the spleen.

Group	Control	AR	Intravenous CD40 siRNA	Nasal CD40 siRNA
DCs (%)	69.150 (65.950-75.925)	84.750 (73.275-88.725)	73.450 (70.400-84.375)	75.850 (69.550-84.625)
CD40^+^ DCs (%)	15.818 (9.232-22.754)	33.087 (21.737-38.601)	16.121 (10.149-27.951)	15.539 (9.273-29.475)
Tregs (%)	4.803 (4.249-7.042)	0.687 (0.373-3.035)	2.075 (1.490-2.814)	3.728 (2.142-4.289)

**Table 2:** Histopathology results of tissue remodelling.

Group	Control	AR	Intravenous CD40 siRNA	Nasal CD40 siRNA
PAS staining (goblet cells number)	75.70 (35.75-88.25)	55.00 (42.50-98.75)	73.00 (51.50-109.50)	53.00 (41.50-65.50)
MT staining (percentage of collagen deposition)	7.269 (6.429-8.285)	19.369 (15.760-21.596)	8.521 (7.565-10.088)	9.306 (8.305-10.247)

## Data Availability

The datasets used and/or analysed during the current study are available from the corresponding author on reasonable request.
